# Impact of resuscitation-trained healthcare workforce availability on neonatal asphyxia mortality: a population-based study

**DOI:** 10.1016/j.resplu.2026.101260

**Published:** 2026-02-10

**Authors:** Mandira D. Kawakami, Adriana Sanudo, Ana Sílvia S. Marinonio, Kelsy N. Areco, Rita de Cássia X. Balda, Milton H. Miyoshi, Daniela T. Costa-Nobre, Tulio Konstantyner, Carina N. Vieira e Oliveira, Paulo Bandiera-Paiva, Rosa M.V. Freitas, Mônica L.P. Teixeira, Bernadette Waldvogel, Carlos Roberto V. Kiffer, Maria Fernanda de Almeida, Ruth Guinsburg

**Affiliations:** a Escola Paulista de Medicina – Universidade Federal de São Paulo, Brazil; b Fundação Sistema Estadual de Análise de Dados, Brazil

**Keywords:** Neonatal mortality, Perinatal asphyxia, Resuscitation, Newborn infant, Healthcare professionals

## Abstract

**Background:**

Intrapartum events cause approximately 900,000 neonatal deaths annually worldwide. This study investigated whether the number of healthcare providers trained in resuscitation at the municipal level is associated with decreased neonatal mortality due to perinatal asphyxia.

**Methods:**

This population-based study analyzed neonatal deaths with birth weight ≥1500 g without congenital anomalies in São Paulo State, Brazil (2011–2020). Deaths were classified as asphyxia-associated when ICD-10 codes indicating intrauterine hypoxia, birth asphyxia, or neonatal meconium aspiration appeared on any line of death certificates. Temporal trends of neonatal mortality rate associated with perinatal asphyxia (Asphyxia-NMR) and density of Brazilian Neonatal Resuscitation Program (NRP) trained professionals per thousand live births across the 645 municipalities of the State were analyzed using Prais-Winsten regression. A multilevel mixed-effects logistic regression included three hierarchical levels (newborns, municipalities, regional health districts), adjusting for maternal age, prenatal visits, delivery mode, newborn sex, NRP-trained professional density, Gross Domestic Product (log-transformed), and year of birth.

**Results:**

Among 6,044,527 live births, 2527 neonatal deaths met inclusion criteria. Asphyxia-NMR declined from 0.43‰ (2011) to 0.31‰ (2020), an annual reduction of 3.84% (95% CI: 0.46–7.10%). The density of NRP-trained professionals rose from 1.67‰ to 35.78‰, an annual increase of 39.97% (95%CI: 24.50–57.35%). Having ≥7 trained professionals per thousand live births decreased the odds of asphyxia-associated neonatal deaths (OR 0.88; 95%CI: 0.80–0.97).

**Conclusion:**

Greater availability of healthcare professionals trained in neonatal resuscitation was independently associated with lower neonatal mortality associated with asphyxia, after adjustment for biological, economic, and regional factors.

## Highlights

• Number of NRP trained providers to reduce perinatal asphyxia deaths is unknown.
• Municipalities with ≥7 trained providers/1000 births had 12% fewer asphyxia deaths.
• Data may guide strategies to reduce preventable neonatal asphyxia deaths.

## Introduction

The neonatal period accounts for 47% of deaths occurring within the first five years of life. The leading causes of neonatal mortality are prematurity, intrapartum complications (including birth asphyxia and trauma), infections, and congenital anomalies.^1^ Birth asphyxia, defined as the failure to establish breathing at birth, accounts for an estimated 900,000 deaths annually worldwide.^2^ In Brazil, a population-based study reported 35,443 early neonatal deaths associated with perinatal asphyxia among neonates ≥37 weeks of gestation without congenital anomalies between 2000 and 2020.^3^ Intrapartum-related deaths can be reduced by up to 70% through adequate intrapartum management and by an additional 22% through neonatal resuscitation.^4^

Neonatal resuscitation is required for approximately 5% of term neonates with respiratory depression at birth, primarily through positive pressure ventilation.^5^ Training healthcare providers in neonatal resuscitation is a cornerstone of developing a structured and effective health system for neonatal care immediately after birth.^6^, ^7^

Several studies have evaluated the impact of neonatal resuscitation training on reducing early neonatal mortality in low-income countries with high neonatal death rates. A meta-analysis including 341,502 newborns demonstrated that immediate assessment at birth combined with face-mask positive pressure ventilation reduced early neonatal mortality by 38%.^8^ In Tanzania and Nepal, mortality within the first 24 h after birth was reduced by nearly 50% following implementation of the Helping Babies Breathe program.^9^, ^10^ Another meta-analysis, encompassing 1,653,805 births in low- and middle-income countries, reported 42% reduction in first-day mortality and 18% reduction within the first week after a neonatal resuscitation training program.^11^ The importance of having at least one team member trained in an accredited advanced life support course was also demonstrated in a systematic review of 14 studies conducted in low- and middle-resource settings, which showed a 22% reduction in early neonatal mortality (RR 0.78; 95% CI 0.63–0.97) among 296,300 neonates.^12^

In this context, the optimal number of healthcare professionals trained in neonatal resuscitation needed to impact neonatal mortality associated with perinatal asphyxia remains unclear. The present study aims to investigate whether a minimum number of trained healthcare providers at the municipal level is associated with a decrease in the neonatal mortality associated with perinatal asphyxia (Asphyxia-NMR).

## Methods

This population-based study included live births from 0 to 27 days of age, with birth weight ≥1500 g and without congenital anomalies in São Paulo State, Brazil, between January/2011 and December/2020. São Paulo State, located in the southeastern region of Brazil had over 44 million inhabitants in 2022, a Human Development Index of 0.806, and an annual gross domestic product (GDP) per capita of US$12,729.37.^13^, ^14^

Data was extracted from the Civil Registry Information, which covers 99.7% of livebirths and 99.8% of deaths in the state,^15^ provided by the Statistical Bureau of the State of São Paulo (Fundação SEADE). Fundação SEADE provided two sets of data: information on all liveborn infants based on live birth certificates from 2011 to 2020; information on all infant deaths from death certificates linked to live birth certificates by deterministic linkage, as previously detailed.^16^

Neonatal deaths (0–27 days) were considered as associated with perinatal asphyxia according to the International Classification of Diseases, 10th revision (ICD-10), described in any line of death certificate as P20.0 (Intrauterine hypoxia first noted before onset of labor), P20.1 (Intrauterine hypoxia first noted during labor and delivery), P20.9 (Intrauterine hypoxia, unspecified), P21.0 (Severe birth asphyxia), P21.1 (Mild and moderate birth asphyxia), P21.9 (Birth asphyxia, unspecified), or P24.0 (Neonatal aspiration of meconium).^17^ Live births were excluded if codes Q00–Q99 (congenital malformations, deformations and chromosomal abnormalities)^17^ were reported on the live birth and/or death certificates.

The live birth database provided maternal variables (municipality of residence, age, number of prenatal care visits, type of pregnancy, place of birth, delivery mode, and gestational age) and neonatal variables (municipality, date, year, and time of birth, birth weight, and sex). The death database included information on municipality of birth and death, age at death, and all causes recorded on the death certificate. Infants were classified by municipality and regional health district of birth. In Brazil, municipalities are the smallest administrative units and the primary level at which health, demographic, and socioeconomic data are collected. They are responsible for organizing primary healthcare and coordinating access to higher levels of care within a mixed public–private system under the Brazilian Unified Health System. In São Paulo State, which comprises 645 municipalities,^18^ public, philanthropic, and private hospitals (often contracted by the public system) play an integral role in municipal and regional maternal and neonatal care networks. São Paulo State is administratively divided into 18 regional health districts, each grouping multiple municipalities and coordinating regional healthcare planning and referral networks.

The Neonatal Resuscitation Program of the Brazilian Society of Pediatrics (BNRP), established in Brazil in 1994, comprises four hands-on courses: *Neonatal Resuscitation for Infants ≥34 Weeks, Neonatal Resuscitation for Preterm Infants <34 Weeks, Neonatal Transport, and Neonatal Resuscitation for Traditional Birth Attendants*. The course for infants ≥34 weeks is an 8-h program that includes a pre-test, two didactic sessions, five hands-on stations addressing initial steps, positive pressure ventilation, tracheal intubation, chest compressions, and medication administration, and a post-test. Each hands-on session is conducted with one instructor per six to eight healthcare professionals. Since 2011, the BNRP has maintained an electronic database of all healthcare professionals trained and certified in this course.^19^ For this study, an anonymous database with the number of healthcare professionals that completed the course from 2011 to 2020 in São Paulo State, Brazil, was provided, according to their municipality of residence and year of training.

The socioeconomic variables selected for the study were those available annually from 2011 to 2020: per capita Public Health Expenditure (PHE) and GDP. Per capita PHE was calculated by dividing the total municipal health expenditure from all funding sources by the resident population.^20^ The GDP was the total value of all goods and services produced in each municipality.^21^

## Statistical methods

The study was divided into two periods (2011–2015 and 2016–2020), corresponding to updates in the BNRP guidelines. Asphyxia-NMR was calculated as follows: −Numerator: number of live births ≥1500 g without congenital anomalies who died with perinatal asphyxia between 0 and 27 days after birth in each municipality of São Paulo State; −Denominator: number of live births in each municipality. Data were organized by municipality, regional health district, and year of birth.

The number of trained healthcare professionals by BNRP was calculated by the cumulative number of certified healthcare professionals by municipality of residence, with an attrition rate of 10% per year in first period, and 20% per year in second period. Workforce attrition was considered due to gradual and expected reduction of active professionals working in the delivery room. Annual attrition rates around 5–10% have been consistently reported in studies evaluating healthcare workforce retention, particularly among nursing, midwifery and maternal-child health professionals, reflecting retirement, migration, career change, and reduced clinical activity over time.^22^ For the second period, a higher attrition rate (20% per year) was applied to account not only for natural workforce loss, but also for the expected overlap between professionals trained in the first and second periods. This approach aims to reduce the risk of overestimating the active workforce by acknowledging partial duplication and diminishing marginal returns of cumulative training programs over time.^23^ The density of BNRP-trained healthcare professionals was calculated as the cumulative number of BNRP-trained healthcare professionals by municipality divided by the number of live births per municipality in each period and expressed per thousand live births. Data were organized by municipality, regional health district, and year of birth. More granular system- or facility-level information was not available in the dataset provided by Fundação SEADE in order to comply with Brazilian data protection regulations*.*

The temporal trend of Asphyxia-NMR and density of BNRP-trained healthcare professionals in all 645 municipalities of São Paulo State were analyzed by Prais-Winsten model, expressed by the annual percentage change and its 95% confidence interval (95%CI).

Pearson’s correlation coefficient (*r*), along with its 95%CI and the coefficient of determination (*R*^2^), was used to analyze the strength and the direction of the linear association between Asphyxia-NMR and density of BNRP trained healthcare professionals, as well as between Asphyxia-NMR and per capita PHE, expressed in Brazilian Reais, for São Paulo State annually, over the entire study period.

To assess the association between the presence of BNRP trained health professionals and neonatal deaths with asphyxia, a multilevel mixed-effects logistic regression model was fitted with three hierarchical levels: (level 1), nested within municipalities (level 2), which were nested within regional health districts (level 3). The dependent variable was the occurrence of neonatal deaths with asphyxia (binary outcome). Independent variables included biological factors (maternal age, number of prenatal care visits, delivery mode, and neonatal sex) and contextual factors (density of BNRP-trained healthcare professionals and log-transformed GDP), as well as the period of study (2011–2015 vs. 2016–2020). The density of BNRP trained healthcare professionals was expressed as a binary variable with the cutoff of 7 per thousand livebirths based on the median number of healthcare professionals trained in the 645 municipalities during the study period. Random intercepts were included at both municipality and regional health district levels to account for unobserved heterogeneity. Odds ratios (OR) and respective 95%CI were reported. Model fit was evaluated using likelihood-ratio tests comparing the multilevel model with a standard logistic regression model. The model was estimated using adaptive Gauss-Hermite quadrature with 7 integration points, as implemented by the “*melogit”* command in StataNow/MP 19.5 (StataCorp LLC, College Station, TX, USA).

Spatial analysis was applied to evaluate the distribution of the crude Asphyxia-NMR and the density of BNRP-trained healthcare professionals by the 645 São Paulo State municipalities in both periods, and to explore spatial autocorrelation by visual effect. Smoothing techniques could not be applied due to the rare occurrence of both variables in the spatial distribution. Thematic maps were developed by TerraView 4.2.2 software (INPE, São José dos Campos, Brazil).

The study was approved by the Ethics Committee on Human Research of Universidade Federal de São Paulo, by Fundação SEADE and the Brazilian Society of Pediatrics.

## Results

**Fig. 1:**
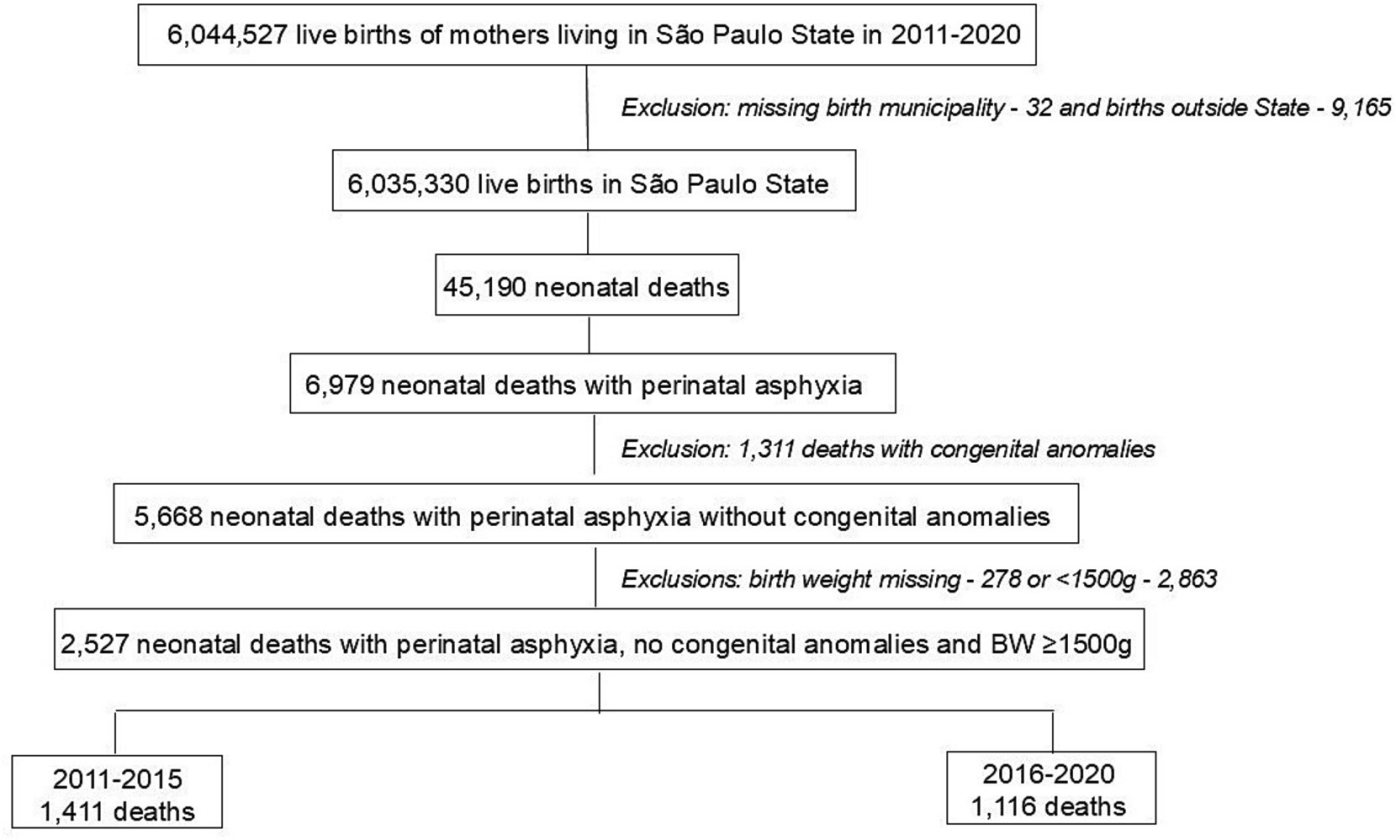
**Flow diagram of the included population**.

From 2011 to 2020, a total of 6,035,330 live births met the inclusion criteria, of which 45,190 died in the neonatal period. Among these deaths, 6979 (15%) were associated with perinatal asphyxia. Of those, 2527 occurred in newborns without congenital anomalies and with a birthweight ≥1500 g (Fig. 1).

**Fig. 2:**
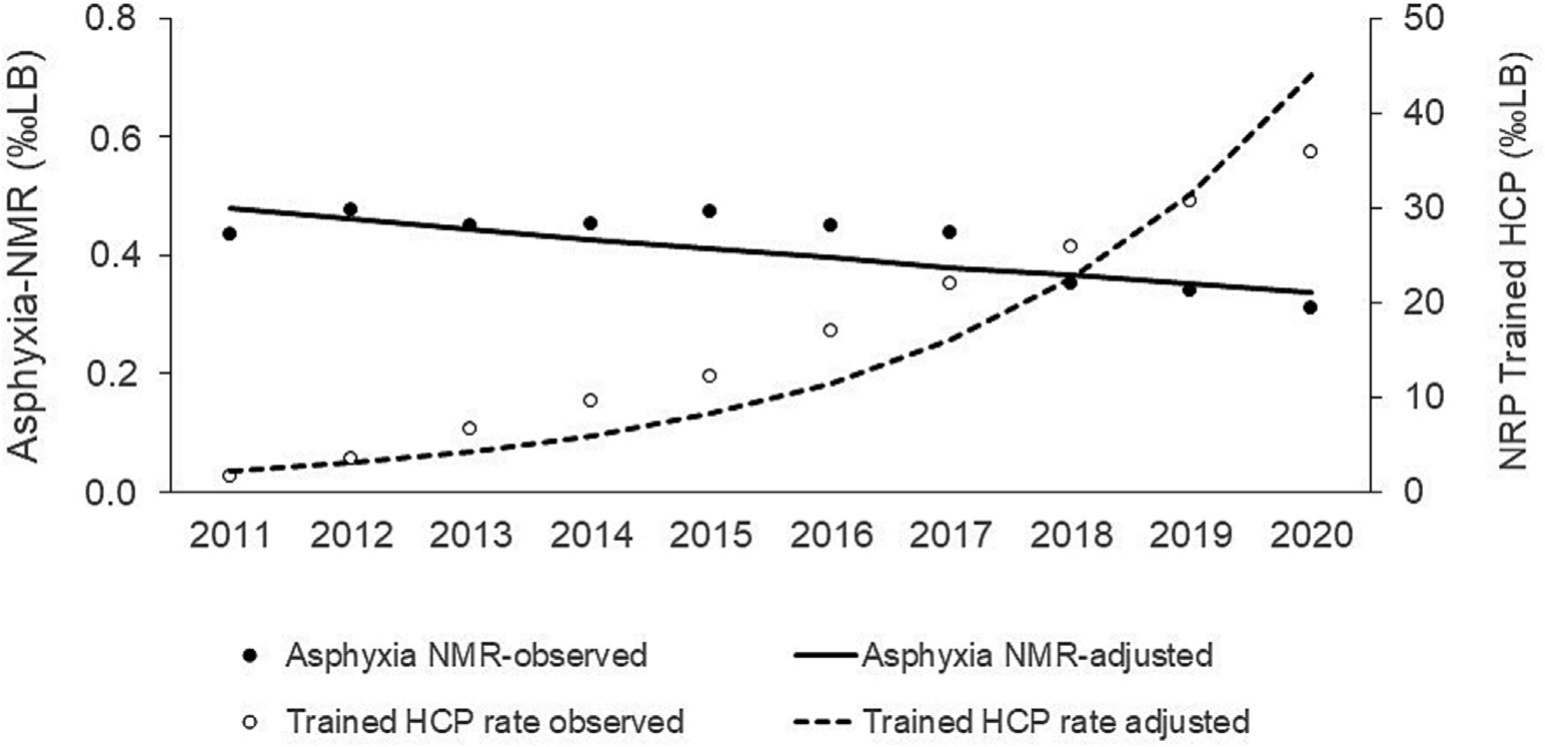
**Temporal trend of neonatal mortality rate with perinatal asphyxia (Asphyxia NMR) per thousand live births (LB) and density of Brazilian Neonatal Resuscitation Program trained healthcare professionals (NRP trained HCP) per thousand live births in São Paulo State, Brazil, from 2011 to 2020, based on Prais-Winsten modeling**.

Maternal and neonatal characteristics of included infants that died with perinatal asphyxia is shown in Table 1. The Asphyxia-NMR decreased from 0.43‰ in 2011 to 0.31‰ live births in 2020 (Table 2), with an annual percent decrease of 3.84% (95% CI: 0.46–7.10%) (Fig. 2). BNRP-trained healthcare professionals density increased from 1.67‰ in 2011 to 35.78‰ live births in 2020 (Table 2), with an annual percent increase of 39.97% (95%CI: 24.50–57.35%) (Fig. 2).

**Table 1:** Maternal and neonatal characteristics of neonatal deaths with perinatal asphyxia without congenital anomalies and birth weight ≥1500 g.

**Characteristics**	**2011–2015**	**2016–2020**
Maternal age (years)	*n* = 1411*	*n* = 1116*
<20	256 (18%)	145 (13%)
20–34	889 (63%)	751 (67%)
≥35	266 (19%)	220 (20%)

Number of prenatal visits	*n* = 1362*	*n* = 756*
0	60 (5%)	17 (2%)
1–6	427 (31%)	220 (29%)
≥7	875 (64%)	519 (69%)

Type of pregnancy	*n* = 1411*	*n* = 1116*
Singleton	1371 (97%)	1089 (98%)

Place of birth	*n* = 1411*	*n* = 1115*
Birth at hospital	1391 (99%)	1095 (98%)

Type of delivery	*n* = 1411*	*n* = 1105*
Cesarean	895 (63%)	694 (63%)

Gestational age (weeks)	*n* = 1385*	*n* = 779*
<32	85 (6%)	49 (7%)
32–36	351 (25%)	181 (23%)
37–41	922 (67%)	529 (68%)
≥42	27 (2%)	20 (2%)

Birth weight (grams; mean ± SD)	2901 ± 732	2902 ± 709

Sex	*n* = 1411*	*n* = 1116*
Male	786 (56%)	612 (55%)

* Number of deaths with information available; SD: standard deviation.

**Table 2:** Annual rate of Neonatal Mortality Associated with Asphyxia, Brazilian Neonatal Resuscitation Program trained healthcare professionals and Public Health Expenditure per capita (in Brazilian Reais) in São Paulo State, Brazil, from 2011 to 2020.

**Year of birth**	**Live births**	Neonatal deaths with asphyxia*	**Asphyxia NMR (‰ LB)**	**Brazilian NRP trained HCP**	**Cumulative number of NRP trained HCP**	**Trained HCP rate (‰ LB)**	**PHE per capita (Brazilian Reais)**
2011	609,713	264	0.43	1131	1018	1.67	553
2012	616,116	293	0.48	1136	2154	3.49	651
2013	610,211	274	0.45	1973	4042	6.62	700
2014	624,663	282	0.45	2159	6195	9.63	820
2015	631,756	298	0.47	1484	7702	12.18	812
2016	599,368	270	0.45	2973	10,195	16.98	866
2017	610,073	267	0.44	3147	13,386	21.90	898
2018	604,489	213	0.35	2311	15,659	25.85	1011
2019	579,730	196	0.34	2721	18,415	30.70	1060
2020	549,211	170	0.31	1083	19,691	35.78	1185

* Infants ≥1500 g without congenital anomalies; HCP: healthcare professionals; LB: live births; NMR: neonatal mortality rate; NRP: Neonatal Resuscitation Program; PHE: Public Health Expenditure.

**Fig. 3:**
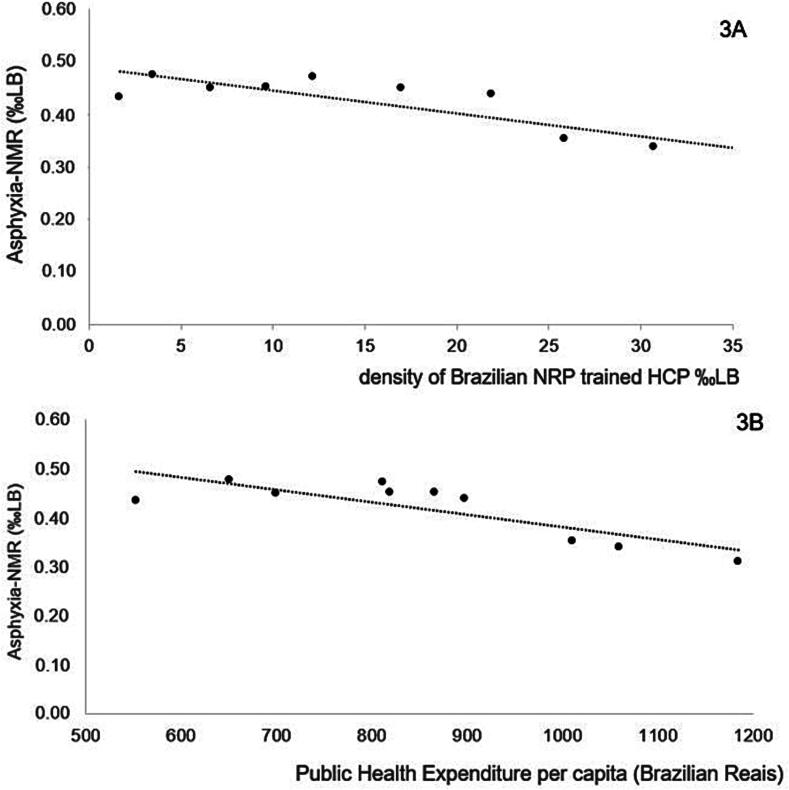
**Pearson linear correlations of neonatal mortality rate with perinatal asphyxia (Asphyxia-NMR) per thousand live births (LB) from 2011 to 2020 in São Paulo State, Brazil, with variables of interest. Panel 3A – Correlation with the density of Brazilian Neonatal Resuscitation Program trained healthcare professionals (NRP trained HCP). Panel 3B – Correlation with Public Health Expenditure per capita**.

Fig. 3A shows a significant negative correlation between Asphyxia-NMR and the density of BNRP-trained healthcare professionals per thousand live births (*r* = −0.860; 95%CI: −0.966 to −0.503) in São Paulo State from 2011 to 2020. The density of BNRP-trained professionals explained 74% of the total variability of Asphyxia-NMR (*R*^2^ = 0.74). Asphyxia-NMR had also a negative correlation with per capita PHE, as shown in Fig. 3B (*r* = −0.820; 95%CI: −0.956 to −0.395). PHE explained 67% of the total variability of Asphyxia-NMR (*R*^2^ = 0.67).

The multilevel mixed-effects logistic regression analysis identified biological and contextual factors significantly associated with the odds of neonatal death due to perinatal asphyxia. A total of 5,591,654 live births with complete data was included in the final model and the results are shown in Table 3. Density of healthcare professionals ≥7 per thousand livebirths trained in BNRP was associated with a reduced odd of 12% in neonatal deaths due to perinatal asphyxia in infants with birthweight ≥1500 g without congenital anomalies (Table 3). The model included random intercepts at the municipality and health district levels to account for unobserved heterogeneity. The estimated variance at the municipality level was 0.057 (95%CI: 0.030–0.111), and at the health district level, 0.076 (95%CI: 0.034–0.170). These values indicate that part of the variability in neonatal mortality associated with perinatal asphyxia remains explained by geographical clustering, beyond what is accounted for by the fixed effects. A likelihood-ratio test comparing the multilevel model to a standard logistic regression confirmed that the multilevel specification significantly improved model fit (Likelihood Ratio test 150.6; *p* < 0.001), supporting the presence of contextual clustering.

**Table 3:** Results from multilevel mixed logistic regression for the associations between the different studied factors and neonatal deaths with asphyxia in livebirths with birth weight ≥1500 g without congenital anomalies in São Paulo State, Brazil, 2011–2020.

**Variables**	**Odds ratio**	**95%CI**	***p*-value**
**Maternal age (years)**			
<20 vs. 20–34	1.30	1.16–1.45	<0.001
≥35 vs. 20–34	1.32	1.20–1.47	<0.001
Cesarean section vs. vaginal birth	1.27	1.17–1.38	<0.001
Male vs. female	1.18	1.09–1.28	<0.001
Density of NRP-trained HCP ≥7/1000 LB vs. <7/1000LB	0.88	0.80–0.97	0.010
log-transformed GDP	0.92	0.88–0.97	0.001
Period 2016–2020 vs. 2011–2015	0.98	0.91–1.07	0.726

CI: confidence interval; GDP: Gross Domestic Product; HCP: healthcare professionals; LB: live births.

**Fig. 4:**
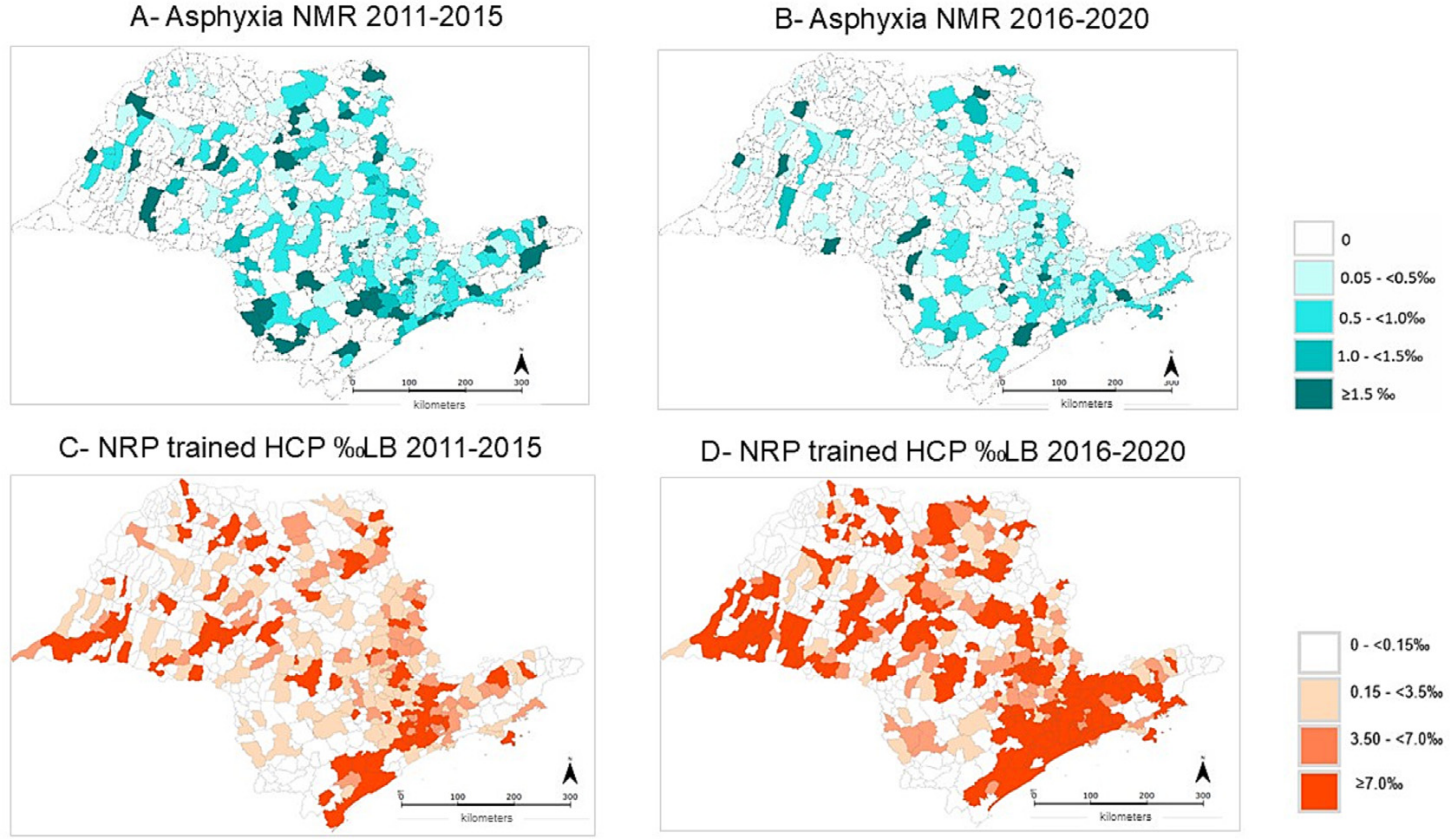
**Spatial distribution of the neonatal mortality rate with perinatal asphyxia (Asphyxia-NMR) per thousand live births in 2011–2015 (Panel A) and 2016–2020 (Panel B) and density of Brazilian Neonatal Resuscitation Program trained healthcare professionals (NRP trained HCP) per thousand live births (LB) in 2011–2015 (Panel C) and 2016–2020 (Panel D) in the 645 municipalities of São Paulo State, Brazil**.

Additionally, Fig. 4A shows that Asphyxia-NMR ≥1.00‰ live births was present in 61 municipalities in 2011–2015 and decreased to 28 municipalities in 2016–2020. Fig. 4B shows that the number of municipalities in São Paulo State with BNRP-trained healthcare professionals ≥7‰ live births rose from 92 (2011–2015) to 194 (2016–2020) municipalities.

## Discussion

This is the first study to demonstrate that the presence of ≥7 healthcare professionals per thousand live births trained in neonatal resuscitation is associated with a reduction in 12% of neonatal deaths with asphyxia in infants with birth weight ≥1500 g without congenital anomalies. During the study period, the Asphyxia NMR reduced 3.84% per year in this population, while the rate of BNRP-trained healthcare professionals increased 39.97% per year, with a strong correlation between these two indicators of quality in neonatal care.

Over the past three decades, substantial global efforts have been directed toward reducing preventable neonatal mortality, guided by initiatives such as the Millennium Development Goals and the Sustainable Development Goals, which set a target neonatal mortality rate of ≤12‰ live births by 2030 for all countries.^24^ As part of strategies to address infant mortality, resuscitation training has been widely implemented through educational programs such as Helping Babies Breathe, which has been associated with reductions in neonatal mortality, particularly in the first day after birth.^25^ Our findings further underscore the critical role of skilled healthcare professionals at birth, demonstrating that resuscitation training is robustly associated with reduced neonatal mortality from asphyxia, even after adjustment for biological risk factors and economic indicators, considering the municipality and the health district of maternal residence. The use of a multilevel mixed-effects logistic regression model with three hierarchical levels was essential to appropriately capture the complex structure of the data. This modeling approach accounts for both individual-level and contextual-level influences on neonatal mortality associated with asphyxia, while also addressing the non-independence of observations within geographic clusters. By incorporating random intercepts at municipality and health districts levels, the model effectively adjusted for unmeasured area-level confounding and allowed for more accurate estimation of fixed effects. Moreover, the adoption of adaptive Gauss-Hermite quadrature (7 points) ensured precise estimation of model parameters, even under complex random-effects structures. These methodological choices enhanced the stability and interpretability of the observed associations. The analytical model sought to capture the complexity of individual and contextual factors influencing neonatal mortality due to perinatal asphyxia and demonstrated that, within this network of determinants, BNRP training of healthcare professionals is associated with a protective effect.

In our study, approximately 67% of mothers whose newborns died from perinatal asphyxia had attended at least seven prenatal visits, a proportion lower than the 79% observed among all 6,016,072 live births in São Paulo State between 2011 and 2020.^26^ This gap may reflect difficulties in identifying risk factors during antenatal care, as well as insufficient communication with families about risk situations that would justify closer monitoring of fetal well-being and early interventions to prevent complications of perinatal asphyxia.^27^

Nearly all (98.5%) births of newborns ≥1500 g who died from perinatal asphyxia in our study occurred in hospitals, reflecting the well-established health system infrastructure in São Paulo State. The cesarean section rate of 63% likely reflects emergency obstetric interventions in response to fetal risk related to asphyxia. Yet, progression to death suggests potential delays in performing these procedures, possibly due to limited and unequal access to timely care. Supporting this concern, a study in Northeast Brazil reported that 29% of 768 pregnant women had to seek care independently across multiple facilities, resulting in delayed management of obstetric emergencies.^28^ Taken together, our findings provide evidence that high coverage of prenatal care and hospital births does not necessarily translate into effective prevention of perinatal asphyxia deaths, underscoring the need for improved risk detection, timely intervention, and equitable access to quality obstetric care.

Our study demonstrates that higher rates of resuscitation trained healthcare professionals explain 74% of the observed variability in neonatal mortality associated with perinatal asphyxia. Neonatal resuscitation training qualifies healthcare professionals attending newborns with low vitality to perform life-saving interventions, such as positive pressure ventilation with face mask. Every second counts for this life-saving procedure as the risk of death increases 16% for every 30 s delay in initiating positive pressure ventilation regardless of the birth weight, gestational age, or complications during gestation or delivery.^29^

In Brazil, neonatal resuscitation training is an integral part of the competency matrix of Brazilian pediatric residency and neonatal fellowship programs.^19^ This broad dissemination is reinforced by federal legislation enacted in 2014, which mandates that all professionals working in delivery rooms of public hospitals and healthcare facilities must be trained in neonatal resuscitation. A central goal of the BNRP is to have at least one certified instructor at each facility with over 500 annual births, tasked with providing ongoing training for the delivery room team and supporting skilled care for every newborn.^19^ The BNRP’s dissemination model likely contributed to the reduction of Asphyxia NMR.^3^

Although neonatal resuscitation equipment and materials are of low technological complexity, they require financial investment for their acquisition and to ensure proper functionality. Consequently, the setup and maintenance of delivery room infrastructure depend on the active involvement and support of healthcare stakeholders in hospitals and maternity facilities.^7^, ^25^ In fact, our study demonstrates that per capita PHE explained 67% of the total variability of Asphyxia-NMR.

It is important to emphasize that the qualification of neonatal teams requires not only the acquisition of technical skills to ensure an organized and competent care at birth, but also proficiency in resuscitation procedures and the ability to function effectively as a team.^30^ This includes effective communication with the obstetric team for the early identification of risk factors for perinatal asphyxia, systematic equipment checks, and clear role assignment during neonatal care. Accordingly, a density of ≥7 trained healthcare professionals in neonatal resuscitation per 1000 live births may serve as a proxy for the presence of qualified birth teams and as a target for public policies aimed at reducing neonatal mortality associated with perinatal asphyxia.

The limitations of this study are mostly related to the use of secondary health data. As the variables were manually extracted from live birth and death certificates, typographical errors and incomplete records due to missing information may have been introduced. Moreover, not all variables included in the certificates were incorporated into the computerized database, limiting the number of valid observations. It should be noted that the diagnoses were established by the medical team responsible for completing the death certificate, which reinforces the clinical relevance of the reported condition in the fatal outcome, irrespective of whether it was classified as the underlying or immediate cause. The absence of information on the exact hospital of birth and death of the newborns, as well as the precise workplace of trained healthcare professionals, limited a more granular assessment of the association between professional availability and neonatal deaths with perinatal asphyxia. Another limitation is related to the absence of formal sensitivity analyses exploring alternative attrition scenarios. The attrition rates of 10% in the first period and 20% in the second period were adopted as conservative, theory-informed assumptions based on the health workforce planning literature, rather than empirically derived estimates from longitudinal follow-up data. In the statistical analysis of the data, although random intercepts were included to account for unobserved municipal- and district-level heterogeneity, residual confounding due to unmeasured individual and time-varying contextual factors cannot be excluded*.* Finally, the study was restricted to a single state, São Paulo, which has the second highest Human Development Index in Brazil^13^ and the largest number of healthcare professionals trained in neonatal resuscitation nationwide.^19^ Future studies across Brazil are needed to evaluate the external validity of these findings.

## Conclusion

This study demonstrated an association between the number of healthcare professionals trained in neonatal resuscitation per 1000 live births and lower neonatal mortality related to perinatal asphyxia in São Paulo State, Brazil, from 2011 to 2020, after accounting for contextual and biological confounding variables. This finding may inform the planning of targeted public health strategies aimed at reducing this preventable cause of neonatal death, particularly in low- and middle-income countries.

## Authors contributions

MDK, RG and MFBA participated in the concept and design of the study, analysis and interpretation of data, drafting and revising the manuscript. AS participated in concept and design of the study, statistical analysis, interpretation of data, drafting and revising the manuscript. ASSM, DTCN and CRVK participated in concept and design of the study, in the spatial analysis and revised the manuscript. KNA and PBP built the linked database that allowed the analysis of the data and participated in the concept and design of the study, interpretation of data, and revising the manuscript. MHM, RCXB, TK and CNVO participated in concept and design of the study, interpretation of data and revised the manuscript. RMF, MLPT and BW have participated in the concept and design of the study and revising the manuscript. All authors listed on the manuscript approved the submission of this version of the manuscript, take full responsibility for the manuscript and agree to be personally accountable for their own contributions and for ensuring that questions related to the accuracy or integrity of any part of the work, even ones in which the author was not personally involved, are appropriately investigated, resolved, and documented in the literature.

## Institutional review board statement

The study was conducted according to the guidelines of the Declaration of Helsinki and approved by the Ethics Committee of Universidade Federal de São Paulo – UNIFESP, CAAE: 56299822.6.0000.5505.

## Informed consent statement

Informed consent was waived given the retrospective nature of the study and use of unidentified anonymized database.

## CRediT authorship contribution statement

**Mandira D. Kawakami:** Writing – review & editing, Writing – original draft, Methodology, Investigation, Formal analysis, Conceptualization. **Adriana Sanudo:** Writing – review & editing, Writing – original draft, Methodology, Investigation, Formal analysis, Conceptualization. **Ana Sílvia S. Marinonio:** Writing – review & editing, Methodology, Formal analysis, Conceptualization. **Kelsy N. Areco:** Writing – review & editing, Methodology, Formal analysis, Data curation, Conceptualization. **Rita de Cássia X Balda:** Writing – review & editing, Methodology, Investigation, Conceptualization. **Milton H. Miyoshi:** Writing – review & editing, Methodology, Investigation, Conceptualization. **Daniela T. Costa-Nobre:** Writing – review & editing, Methodology, Formal analysis, Conceptualization. **Tulio Konstantyner:** Writing – review & editing, Methodology, Investigation, Conceptualization. **Carina N. Vieira e Oliveira:** Writing – review & editing, Methodology, Investigation, Conceptualization. **Paulo Bandiera-Paiva:** Writing – review & editing, Methodology, Formal analysis, Data curation, Conceptualization. **Rosa M.V. Freitas:** Writing – review & editing, Methodology, Conceptualization. **Mônica L.P. Teixeira:** Writing – review & editing, Methodology, Conceptualization. **Bernadette Waldvogel:** Writing – review & editing, Methodology, Conceptualization. **Carlos Roberto V. Kiffer:** Writing – review & editing, Methodology, Formal analysis, Conceptualization. **Maria Fernanda de Almeida:** Writing – review & editing, Writing – original draft, Methodology, Investigation, Formal analysis, Conceptualization. **Ruth Guinsburg:** Writing – review & editing, Writing – original draft, Methodology, Investigation, Formal analysis, Conceptualization.

## Funding

Fundação de Amparo à Pesquisa do Estado de São Paulo – FAPESP, Project #2017/03748-7. The funding agency did not interfere or participate in the design of the study, data analysis, interpretation of the results, writing or revising of the manuscript.

## Declaration of competing interest

The authors declare that they do not have any conflict of interest. The funding agency (Fapesp) had no role in the design, execution, interpretation, or writing of the study.

## Data Availability

The data are publicly available just in part. The datasets were provided by Fundação SEADE. Requests to access these datasets should be directed to https://produtos.seade.gov.br/produtos/mrc/. The database with linked information of birth and death certificates of São Paulo State, from 2011 to 2020, is available on request with the corresponding author.
